# The Impact of Localized Muscle Fatigue on Multi-Joint Biomechanical Strategies During Stair Ascent

**DOI:** 10.3390/life16060898

**Published:** 2026-05-27

**Authors:** Wenyue Ma, Tao Liu, Liangsen Wang, Zhengao Li, Zheng Wang, Yuliang Sun

**Affiliations:** 1 School of Physical Education, Shaanxi Normal University, Xi’an 710119, China; mawnyue@snnu.edu.cn (W.M.); liutao0604@snnu.edu.cn (T.L.); wlsen13@snnu.edu.cn (L.W.); lizhengao@snnu.edu.cn (Z.L.); 2 College of Arts and Sports, Dong-A University, Busan 49315, Republic of Korea; 3 School of Physical Education and Sport Training, Xi’an Physical Education University, Xi’an 710068, China

**Keywords:** localized muscle fatigue, lower-limb biomechanics, compensatory strategy

## Abstract

The objective of this study was to investigate the impact of localized muscle fatigue (LMF) in the hip, knee, and ankle muscle groups on stair ascent biomechanics, with a focus on identifying compensatory mechanisms following fatigue. Twenty-five participants were fatigued using an isokinetic dynamometer to induce unilateral muscle fatigue in the hip extension, knee extension, and ankle plantarflexion muscles through repetitive isokinetic contractions. We collected stair ascent data before fatigue and after three different fatigue protocols, simultaneously collecting kinematic, kinetic, and electromyographic data. The effects of different muscle fatigue conditions on stair ascent performance were assessed using one-way repeated-measures ANOVA. Key findings revealed that hip fatigue narrowed step width and increased ankle dorsiflexion. Knee fatigue reduced knee extensor moments on the fatigued side and increased hip extension moments bilaterally. Ankle fatigue decreased plantar flexion and increased hip extension moments. Electromyographic data confirmed corresponding shifts in muscle activation. Collectively, the results suggest that localized lower-limb fatigue may alter stair ascent biomechanics in a joint-specific manner. The observed changes in joint moments and muscle activation may reflect a bidirectional pattern of inter-joint compensation, with proximal-to-distal or distal-to-proximal adjustments depending on the fatigued joint. These findings suggest that mechanical and neuromuscular demands may be redistributed across lower-limb joints under acute fatigue conditions; however, given that this study was conducted in healthy young males, the relevance of these findings to stair-related instability or fall risk in more vulnerable populations should be examined in future studies.

## 1. Introduction

Stairs are commonly encountered in daily living, yet stair negotiation represents a biomechanically demanding locomotor task that requires precise lower-limb control [1]. Although healthy persons climb stairs quite easily, this movement task becomes more demanding when motor function is reduced. Furthermore, studies indicate that stair incidents are associated with a significantly higher risk of severe injury or death compared to falls on the same level [2,3,4], which can increase the risk of fall-related injuries. This elevated risk is due to the high physical demands of stair ascent. Specifically, ascending stairs differs significantly from walking on level ground as it requires greater muscle strength to propel the body forward and upward [5] and places greater demands on the coordinated control of multiple lower-limb joints.

A key factor that compromises these capacities is fatigue. The study found that muscle fatigue leads to decreased muscle strength, impairs neuromuscular and coordinated control capacities [6,7]. Fatigue is characterized by a transient or sustained decline in the maximal force-producing capability of the neuromuscular system, typically induced by prior activity [7]. This impairment is highly relevant because localized muscle fatigue (LMF) is prevalent in daily life, with evidence indicating that approximately one-third of the U.S. workforce experiences it during work and other daily tasks [8,9]. Prolonged physical exertion can lead to declines in physical capacity and motor control [6,10]. This fatigue-induced impairment subsequently compromises stability, thereby elevating the risk of accidents, injuries, and falls [11]. Muscle fatigue can impair balance and gait quality, increasing the risk of falls [12,13]. In particular, occupationally induced LMF has been identified as an intrinsic factor contributing to slip-and-fall accidents [11].

The effects of lower-limb muscle fatigue on gait have been investigated in previous work. However, gait was mainly investigated during level walking [14]. A study found a relationship between quadriceps LMF and gait parameters associated with a higher risk of slip-induced falls [15]. Research demonstrates that localized quadriceps fatigue compromises the muscle’s force-generating capacity and diminishes knee joint torque throughout the stance phase. These deficits are accompanied by concurrent alterations in lower-limb kinematics, specifically, a reduced ankle plantarflexion angle and an increased knee flexion angle at heel contact [16]. As a result of fatigue in the plantarflexors, the knee flexion angle increases during the loading response phase. Concurrently, reductions are observed in both the knee flexion moment and the ankle plantar flexion moment, with these kinetic alterations typically manifesting at toe-off [17]. Only one study has investigated stair gait post-muscle fatigue. However, the authors selected multi-joint muscle group fatigue for their investigation. They found that lower-limb muscle fatigue compromised stair gait and decreased postural stability during descent, but did not affect gait or postural stability during ascent [18]. Critically, the specific effects of LMF, a prevalent risk factor in slip-and-fall accidents, on stair ascent gait remain unexplored [16]. Therefore, the primary aim of this study is to investigate the impact of LMF on biomechanical parameters during stair ascent.

The present study aimed to investigate the impacts of LMF on the hip, knee, and ankle muscle groups during stair ascent biomechanics, with a focus on uncovering compensatory mechanisms following fatigue. Kinematic, kinetic, and electromyographic (EMG) data were collected before and after fatigue interventions. We hypothesized that localized muscle fatigue at one joint would lead to compensatory adjustments at other joints, with the direction of compensation depending on which joint was fatigued. Specifically, we anticipated that fatigue at a distal joint (ankle) might shift demand proximally, while fatigue at a proximal joint (hip) might lead to distal adjustments. Additionally, we hypothesized that contralateral muscle strength would increase to offset deficits in unilateral muscle strength.

## 2. Materials and Methods

### 2.1. Participants

The sample size was estimated using G*Power software (version 3.1.9.7) based on a one-way repeated-measures ANOVA model (repeated measures, within-factors). The parameters were set as follows: effect size f = 0.25, significance level α = 0.05, statistical power (1 − β) = 0.80, number of repeated measurements = 4, and correlation among repeated measures set to 0.50. The result indicated a minimum required sample size of 24 participants [19].

Based on this calculation, a total of 25 healthy adult males (mean ± SD: age 19.2 ± 1.5 years, body mass 74.3 ± 6.8 kg, height 1.79 ± 0.06 m) were recruited for this study. The exclusion criteria included any neurological or musculoskeletal condition affecting gait or balance, as well as a prior history of lower-limb fracture. Foot dominance was determined as the preferred foot for kicking a ball [20], which was consistently right-sided for all enrolled individuals. The study protocol was approved by the Ethics Committee of the School of Physical Education at Shaanxi Normal University (202516041), and written informed consent was obtained from all participants in accordance with the Declaration of Helsinki.

### 2.2. Apparatus and Procedures

**Figure 1:**
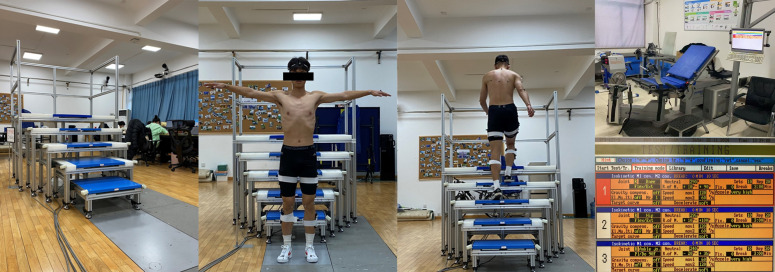
Examples of a staircase, dynamic and static data collection procedures, and a fatigue protocol.

The stair used in this study consists of five steps (Figure 1). The height between steps was 19 cm, and the width was 30 cm, in accordance with Chinese national stair standards [21]. Four force plates (Model 9260AA6, Kistler Instrument Corp., Winterthur, Switzerland; 500 × 600 mm, 1000 Hz) were embedded in the staircase’s first, second, third, and fourth steps to collect ground reaction forces (GRF) data. EMG was recorded using the Delsys Trigno™ Wireless Biofeedback System (Delsys Inc., Boston, MA, USA) at a sampling rate of 2000 Hz. Kinematics were obtained using a Qualisys motion capture system (Oqus700+, Qualisys AB, Gothenburg, Sweden; 200 Hz) with 10 infrared cameras.Kinematic, kinetic, and EMG data were collected simultaneously. Participants underwent four testing sessions in a randomized sequence to control for order effects. Each session featured a distinct stair-climbing trial: one under normal (non-fatigued) conditions and three performed after inducing localized fatigue in the hip extensor, knee extensor, and ankle plantarflexor muscle groups, respectively. A 3-day washout period was maintained between consecutive sessions to ensure full recovery and a return to baseline performance levels. Before the first test, participants were instructed to test their Maximum Voluntary Contraction (MVC) [21]. The tested muscles included Gluteus Maximus, Rectus Femoris, Biceps Femoris, Tibialis Anterior, Medial Gastrocnemius, and Lateral Gastrocnemius [22,23]. Following this, electromyography (EMG) electrodes were applied to these areas. Only the EMG of the dominant leg (right leg) was studied. Fifty-eight reflective markers were placed on each participant at specific anatomical locations [21]. All participants wore standardized apparel and footwear throughout the study. Participants were instructed to complete three valid trials naturally, ensuring the left foot stepped on the first step when ascending the stairs. The remaining trial induced fatigue in the hip extensor, knee extensor, and ankle plantarflexor muscle groups. Each trial followed the same procedure, including a warm-up, fatigue induction, and reflective marker placement. Three valid stair-climbing trials were completed. The interval between muscle fatigue and gait testing should be no longer than 5 min to ensure that participants have not fully recovered, enabling the assessment of fatigue’s effects on gait [24,25].

### 2.3. Fatigue Protocol

Unilateral hip extension, knee extension, and ankle plantarflexion muscle fatigue were induced using an isokinetic dynamometer (IsoMed 2000, D&R Ferstl GmbH, Hemau, Germany) through repetitive isokinetic contractions. The hip and knee extension protocol consisted of concentric–concentric movements at angular velocities of 60°/s (extension) and 120°/s (flexion), while the ankle plantar flexion protocol was performed at 120°/s (plantar flexion) and 60°/s (dorsiflexion) [13,17,26]. The fatigue criteria were determined by examining the participants’ maximum joint torque (MJT) during each exercise. We set twenty repetitions per set, ten sets in total, and a 3 min rest interval between sets. For each movement, participants were instructed to exert maximum effort. Once three consecutive repetitions fell below 50% of the MJT, fatigue was considered to have occurred [24].

### 2.4. Movement Phase Definition

**Figure 2:**
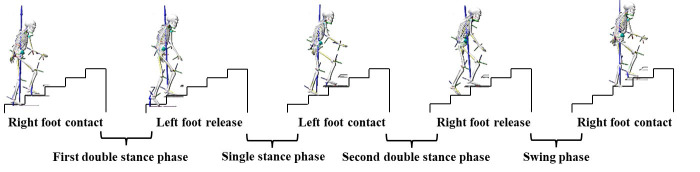
Gait cycle phase division on the fatigued side.

The stair configuration consisted of five steps. Gait events, specifically foot contact and foot release, were instrumented using Visual 3D software (V6.0, C-Motion, Germantown, MD, USA), with a 10 N threshold for the vertical ground reaction force [21]. A complete gait cycle was defined as the interval from the initial contact of the right foot on the second step (0% of the cycle) to its subsequent contact on the fourth step (100% of the cycle). This cycle was demarcated by four key events: right-foot contact (second step), left-foot release (first step), left-foot contact (third step), and right-foot release (second step) Figure 2. These events sequentially initiated the first double-stance phase, the single-stance phase, the second double-stance phase, and the swing phase. The steps were numbered from the bottom as Step 1 to Step 5.

### 2.5. Data Analysis

The independent variable in this experiment was the fatigue protocol (Pre-fatigue, hip extension muscle fatigue, knee extension muscle fatigue, and ankle plantarflexion muscle fatigue). The dependent variables consisted of various kinematic and kinetic gait parameters. The data extracted as C3D files were imported into Visual 3D for further processing. Kinematic and kinetic data were filtered using a fourth-order Butterworth low-pass digital filter with cutoff frequencies of 14 Hz for kinematics and 50 Hz for kinetics [21,27]. An X-Y-Z Cardan sequence, defined according to the right-hand rule relative to the segment coordinate axes, was used for rotation order [21]. The EMG signals were removed from the mean value and analyzed after band-pass filtering (zero-phase fourth-order Butterworth filter, 20–500 Hz) [28]. For each muscle, smoothed EMG magnitudes were normalized to the MVC [21]. We calculated the root-mean-square (RMS) for each phase. Lower limb sagittal joint moments were calculated using inverse dynamics and normalized to 100% of the gait cycle duration.

### 2.6. Statistical Analysis

Statistical analyses were performed using MATLAB (R2018b; The MathWorks Inc., Natick, MA, USA) and SPSS (version 25; IBM Corp., Armonk, NY, USA). Scalar variables, including spatiotemporal parameters and electromyography (EMG) signals, were first examined for normality. Subsequently, one-way repeated-measures ANOVA was conducted to examine the effects of different fatigue conditions (pre-fatigue, post-hip extensor fatigue, post-knee extensor fatigue, and post-ankle plantarflexor fatigue) on scalar outcome measures. Data normality was assessed using the Shapiro–Wilk test, and sphericity was evaluated using Mauchly’s test. When the assumption of sphericity was violated, Greenhouse–Geisser corrections were applied.

When significant main effects were identified, post hoc pairwise comparisons based on Estimated Marginal Means (EMMeans) were performed. Bonferroni adjustment was applied within each ANOVA model to control for multiple pairwise comparisons. Partial eta squared (ηp^2^) was reported as the measure of effect size [29].

To further control for false discovery across multiple outcome domains (spatiotemporal and EMG variables), *p*-values from post hoc comparisons were additionally adjusted using the Benjamini–Hochberg False Discovery Rate (FDR) procedure (q = 0.05), applied across all post hoc comparisons within each outcome domain (spatiotemporal and EMG variables).

For continuous waveform variables (joint angles and joint moments), one-way repeated-measures ANOVA implemented in the SPM1d toolbox was used to assess condition effects across the entire movement cycle [30]. Because SPM1d uses random field theory-based cluster-level inference, no additional FDR correction was applied. Statistical significance was set at *p* < 0.05 for all analyses.

## 3. Results

### 3.1. Verification of Fatigue Intervention

All participants completed the experiment, and following each fatigue intervention, the targeted muscle group demonstrated at least a 50% decrease in its flexor or extensor MJT. Differences in fatigue rates were observed among different muscle groups based on the number of fatigue intervention sets and maximum joint torque data. Larger joint muscle groups (knee extensors) demonstrated greater fatigue resistance, whereas smaller joint muscle groups (ankle plantarflexors) fatigued more rapidly.

### 3.2. Spatiotemporal Parameters

**Figure 3:**
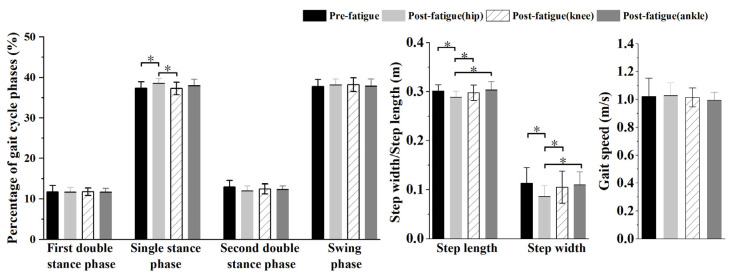
Comparison of spatiotemporal parameters pre- and post-fatigue. Results are presented by phases, with each phase displaying four test conditions from left to right: pre-fatigue, post-hip extensor fatigue, post-knee extensor fatigue, and post-ankle plantarflexor fatigue. * Indicates a significant difference (*p* < 0.05) relative to the pre-fatigue condition.

The one-way repeated-measures ANOVA revealed significant main effects of the fatigue condition on both the electromyographic (EMG) activity of all recorded muscles and the spatiotemporal parameters. Post hoc planned comparisons (simple contrasts with Bonferroni adjustment) against the pre-fatigue baseline identified specific compensatory adaptations. Specifically, a significant effect was found for step length (F = 3.789, *p* = 0.015, ηp^2^ = 0.166), with post hoc comparisons indicating a reduction following hip extensor fatigue relative to the pre-fatigue baseline (*p* = 0.05). Similarly, step width was significantly affected (F = 4.488, *p* = 0.007, ηp^2^ = 0.191), narrowing substantially after hip extensor fatigue (*p* < 0.001), even though gait speed remained unchanged. Furthermore, the single-support time ratio showed a significant main effect (F = 3.860, *p* = 0.014, ηp^2^ = 0.169), which increased after hip fatigue (*p* = 0.031). No other significant differences were observed in the remaining spatiotemporal parameters (Figure 3).

### 3.3. Muscle Activation Patterns

**Figure 4:**
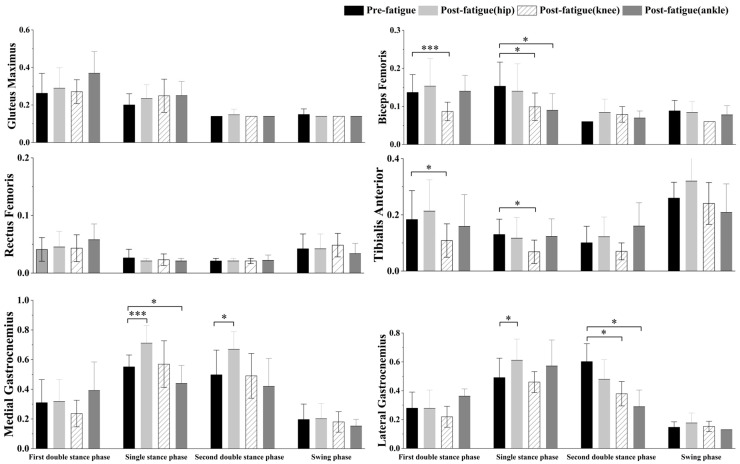
Normalized EMG amplitude (%MVC) during stair ascent under localized fatigue conditions. The normalized EMG amplitude (%MVC) was calculated as the RMS of each muscle’s activity across all phases divided by its corresponding RMS during MVC. Results are presented by phase, with each phase displaying four test conditions from left to right: pre-fatigue; post-fatigue in hip extensors (post-fatigue (hip)); post-fatigue in knee extensors (post-fatigue (knee)); and post-fatigue in ankle plantarflexors (post-fatigue (ankle)). * Indicates a significant difference (* *p* < 0.05); *** indicates a highly significant difference (*** *p* < 0.001).

The one-way repeated-measures ANOVA revealed significant main effects of the fatigue condition on the electromyographic activity of all targeted muscles. Specifically, the lateral gastrocnemius was significantly affected during both single stance (F = 6.237, *p* < 0.001, ηp^2^ = 0.247) and second double stance (F = 31.840, *p* < 0.001, ηp^2^ = 0.626). Post hoc comparisons indicated that its activation was greater following hip extensor fatigue during single stance (*p* = 0.008), but smaller following ankle plantarflexor fatigue during the second double stance (*p* < 0.001). Similarly, the medial gastrocnemius was significantly affected during single stance (F = 19.036, *p* < 0.001, ηp^2^ = 0.500) and second double stance (F = 12.443, *p* = 0.002, ηp^2^ = 0.396), demonstrating greater activation after hip extensor fatigue in both gait phases (*p* < 0.001; *p* = 0.014) and smaller activation after ankle plantarflexors fatigue during single stance (*p* = 0.010). Furthermore, the tibialis anterior (first double stance: F = 17.000, *p* = 0.006, ηp^2^ = 0.508; single stance: F = 5.421, *p* = 0.002, ηp^2^ = 0.222) and the biceps femoris (first double stance: F = 8.009, *p* < 0.001, ηp^2^ = 0.297; single stance: F = 7.080, *p* < 0.001, ηp^2^ = 0.271) both exhibited significantly reduced activation following knee extensor fatigue (all *p* < 0.025), with the biceps femoris also showing smaller activation during single stance after ankle plplantarflexorsatigue (*p* = 0.007). (Figure 4).

### 3.4. Gait Kinematics and Kinetics

We found that post-hip extension muscle fatigue significantly increased the right ankle dorsiflexion angle during the final part of the single-stance (45–50%, *p* = 0.04) and second double-stance (55–60%, *p* = 0.04) phases. No other muscle moment outcomes differed significantly between sides.

Following knee extension muscle fatigue, we demonstrated a decrease in knee extensor moment during the final part of the first double-stance phase (8–12%, *p* = 0.022) and the initial part of the single-stance phase (13–20%, *p* = 0.015). Additionally, we observed a significantly greater hip extension moment during the final part of the second double-stance phase (58–62%, *p* = 0.034) and a decreased hip flexor moment during the initial part of the swing stance (70–75%, *p* = 0.036).

On the no-fatigue side, we demonstrated a significantly greater hip extension during the final part of the first double-stance phase (7–11%, *p* = 0.037). No other angle outcomes differed significantly between sides.

Post-ankle plantarflexion muscle fatigue, we found that on the fatigue side ankle plantar flexion angle significantly decreased during total phase (0–12%, *p* = 0.006; 13–50%, *p* = 0.007; 51–62%, *p* = 0.006; 63–100%, *p* = 0.005); meanwhile, on the no-fatigue side ankle plantar flexion angle decreased during total phase(0–12%, *p* = 0.025; 13–40%, *p* = 0.034; 51–62%, *p* = 0.035; 63–100%, *p* = 0.025).

**Figure 5:**
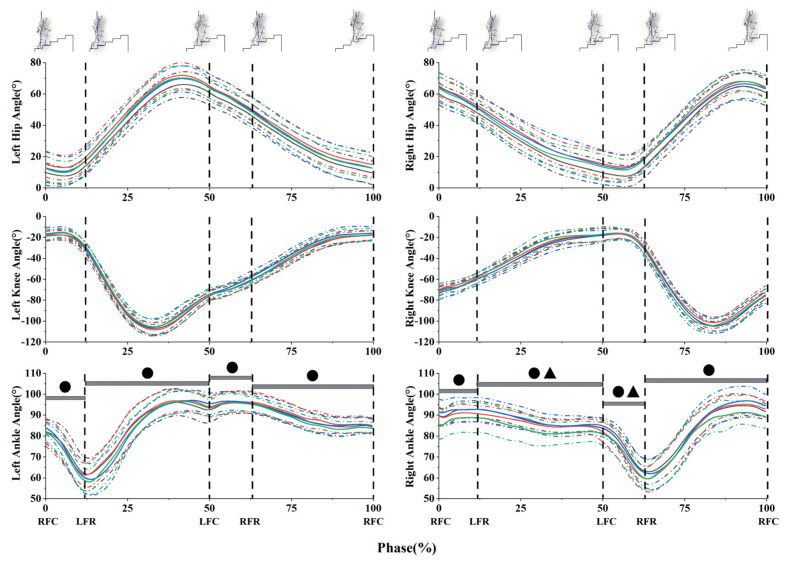

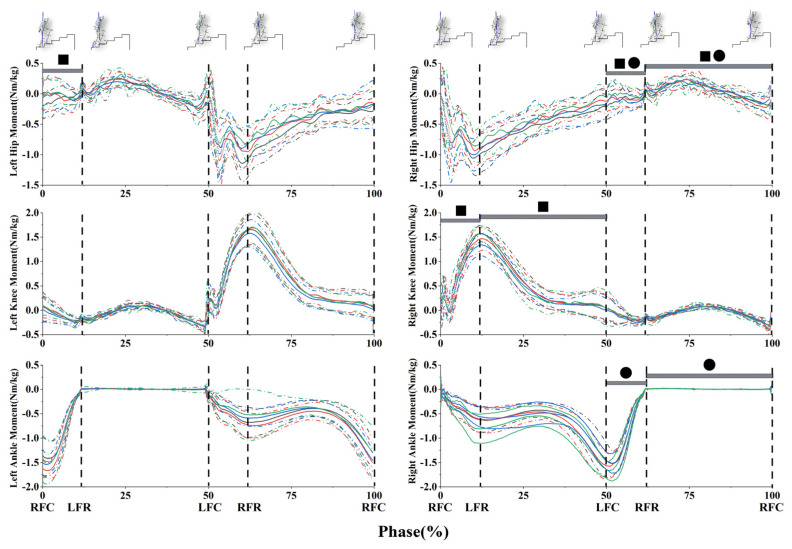
Hip, knee, and ankle angles and moments are in the sagittal plane during stair ascent. The black dashed lines represent gait events: RFC (right-foot contact), LFR (left-foot release), LFC (left-foot contact), and RFR (right-foot release). ● represents post-ankle plantarflexor fatigue; ▲ represents post-hip extension muscle fatigue; ■ represents Post-knee extension muscle fatigue. The black solid line indicates the mean value of the pre-fatigue condition; the red solid line indicates the mean value after fatigue of the hip muscle group; the blue solid line indicates the mean value after fatigue of the knee muscle group; the green solid line indicates the mean value after fatigue of the ankle muscle group; and the dashed lines of the corresponding colors represent the standard deviation.

On the fatigue side, a significantly greater hip extension moment was found during the final part of the double-stance phase (55–62%, *p* = 0.04) and the initial part of the swing phase (63–70%, *p* = 0.003) (Figure 5).

## 4. Discussion

The present study investigated the effects of localized muscle fatigue (LMF) at the hip, knee, and ankle on stair ascent biomechanics, with a particular focus on compensatory strategies. Consistent with our hypothesis, the compensatory patterns depended on the location of the fatigued joint. Specifically, hip extensor fatigue induced greater distal muscular and kinematic adjustments, whereas ankle plantarflexor fatigue shifted mechanical demand proximally toward the hip joint. In addition, unilateral fatigue elicited compensatory changes in both the fatigued and non-fatigued limbs, indicating bilateral coordination adjustments during stair ascent. Although gait speed remained unchanged, localized fatigue altered spatiotemporal parameters, joint mechanics, and muscle activation patterns, supporting the presence of fatigue-location-dependent inter-joint compensation strategies.

(1) Inter-joint Compensatory Strategies.

Our results indicated that joint moments significantly decreased following knee and ankle muscle fatigue. This occurred due to weakened muscular capacity, which diminished joint moment generation, thereby reducing kinetic energy dissipation [31] and impairing landing stabilization [26,32].

Stair climbing is a biomechanically specific activity that requires raising the body’s center of mass (COM) against gravity [5]. This ascent is primarily driven by a coordinated triple extension of the lower limb, comprising hip extension, knee extension, and ankle plantarflexion. The requisite propulsion is generated by major muscle groups, including the gluteus maximus and quadriceps at the hip and knee, and the triceps surae at the ankle, which provides the critical push-off force to elevate the COM [33]. Consequently, the most significant upward movement of the COM occurs during the initial single-stance phase, when this coordinated extension is most critical [34].

From a biomechanical perspective, the direction of compensation may depend on the fatigued joint’s functional role during stair ascent [35,36]. The hip extensors mainly contribute to vertical propulsion, whereas the ankle plantarflexors are more involved in push-off and forward progression [35,36]. Consequently, proximal fatigue may induce distal compensatory adjustments, whereas distal fatigue may shift mechanical demand proximally toward the hip extensors.

Our findings demonstrate that hip extensor fatigue necessitated a distal compensatory strategy. Specifically, we observed increased activation of the medial/lateral gastrocnemius and tibialis anterior muscles during the single- and second-double-stance phases, along with a significant increase in ankle dorsiflexion angle. This pattern may indicate that when proximal hip power generation is compromised, the neuromuscular system modulates ankle control and force distribution to compensate for the substantial deficit in proximal strength and maintain body propulsion.

After knee extensor fatigue, the biceps femoris exhibited significantly reduced activation levels across all movement phases. This observation aligns with previous reports of decreased hamstring activity after quadriceps fatigue during jumping tasks, which has been termed an ‘antagonist inhibition strategy’ [37]. This strategy is considered compensatory, increasing knee mechanical efficiency [38]. A previous study reported reduced peak knee extensor moments after quadriceps fatigue, which aligns with our findings [16]. Additionally, the same study observed greater knee flexion at heel contact and reduced knee extension during terminal stance [16]. However, no such kinematic differences were detected in our investigation. Instead, we observed a compensatory increase in hip extensor moment during the second double-stance phase, indicating that the biomechanical demand was shifted proximally. This finding has not been consistently reported previously, possibly due to differences in fatigue protocols or task demands. When a joint experiences reduced moment output due to fatigue or injury, other joints compensate by increasing their moment generation to maintain overall locomotor stability [39]. On the no-fatigue side, we demonstrated a significantly greater hip extension moment during the first double-stance phase. This compensatory hip extension mechanism preserves forward propulsion when the fatigued side exhibits reduced knee extension capability during gait.

Following ankle plantarflexor fatigue, a significantly greater hip extension moment was observed alongside decreased gastrocnemius activation, indicating a shift in joint kinetics from fatigued to unfatigued muscles [40]. This pattern may reflect a proximal-dominant redistribution of joint kinetics. Given that the ankle plantarflexors are the primary source of push-off force during stair ascent, their fatigue creates a propulsion deficit. Consequently, a greater contribution from proximal muscles may be involved in meeting propulsion demands, particularly the hip extensors, such as the gluteus maximus, to generate the necessary force to elevate the center of mass. This kinetic redistribution is consistent with alterations in lower-limb energy absorption patterns post-fatigue [40]. Notably, decreased plantarflexion angles were observed on the non-fatigued side, suggesting central nervous system-mediated adjustments to preserve gait symmetry [41,42].

This pattern may be interpreted from a broader neuromuscular control perspective. An interesting dissociation between kinetic and kinematic outcomes was observed. The absence of significant angular changes despite altered joint moments suggests that limb trajectories may be relatively preserved under fatigue conditions during stair ascent, a task with high environmental demands and fall risk. Rather than substantial alterations in gross joint kinematics, adjustments appear to be achieved through modulation of muscle forces and joint torques, which may reflect motor redundancy, whereby different combinations of joint torques can preserve similar movement trajectories despite altered muscular demands.

(2) Whole-Body Gait Strategy

A fundamental change in gait strategy was observed at the whole-body level, characterized by a reduction in both step length and width. This adaptation is interpreted as a conservative, stability-oriented strategy. Under fatigue, the neuromuscular system appears to prioritize balance by narrowing the base of support, reducing the amplitude of center-of-mass displacement, and mitigating the increased risk of imbalance associated with diminished muscle strength and compromised joint control.

It should be noted that this study was conducted in an acute laboratory setting involving healthy young males; therefore, the findings should be interpreted as task-specific biomechanical adaptations to fatigue rather than evidence of an underlying neuromuscular control strategy.

This study has several limitations. First, the participants were limited to healthy young adults, partly due to recruitment constraints and partly to minimize the potential fall risk associated with stair ascent in older populations. Therefore, the findings should be interpreted as acute fatigue-related adaptations in healthy young adults and should not be directly generalized to older adults, clinical populations, or individuals with chronic strength deficits. Second, residual fatigue at the time of gait testing was not directly quantified. However, maximal joint torque was substantially reduced immediately after the fatigue protocol; partial recovery during the transition to gait testing cannot be excluded. Third, EMG data were collected only from the dominant limb, so the neuromuscular mechanisms underlying changes in the contralateral joint moment could not be assessed directly.

## 5. Conclusions

In summary, this study suggests that localized muscle fatigue may disrupt lower-limb biomechanics during stair ascent. Under acute laboratory-induced fatigue conditions, the observed joint-specific changes in joint moments and muscle activation may reflect a bidirectional pattern of inter-joint compensation: reduced ankle push-off demand was accompanied by increased hip extension torque, whereas reduced hip power was associated with increased activation of ankle musculature. These findings suggest that kinetic and neuromuscular demands may be redistributed across the lower limb during stair ascent under acute fatigue conditions. The present findings should be interpreted within the context of an acute laboratory fatigue model in healthy young males. They should not be directly generalized to populations with chronic strength deficits or elevated fall risk. Nevertheless, they may provide a useful basis for future studies examining whether similar joint-specific compensatory limitations contribute to stair-related instability in more vulnerable populations.

## Data Availability

The data presented in this study are available from the corresponding author upon reasonable request.

## Abbreviations

The following abbreviations are used in this manuscript:

LMF	Localized Muscle Fatigue
EMG	Electromyography
MVC	Maximum Voluntary Contraction
MJT	Maximum Joint Torque
GRF	Ground Reaction Force
RMS	Root Mean Square
COM	Center of Mass
FDR	False Discovery Rate

## References

[1] Riener R., Rabuffetti M., Frigo C. (2002). Stair Ascent and Descent at Different Inclinations. Gait Posture.

[2] Jacobs J.V. (2016). A Review of Stairway Falls and Stair Negotiation: Lessons Learned and Future Needs to Reduce Injury. Gait Posture.

[3] Startzell J.K., Owens D.A., Mulfinger L.M., Cavanagh P.R. (2000). Stair Negotiation in Older People: A Review. J. Am. Geriatr. Soc..

[4] Svanström L. (1974). Falls on Stairs: An Epidemiological Accident Study. Scand. J. Soc. Med..

[5] McFadyen B.J., Winter D.A. (1988). An Integrated Biomechanical Analysis of Normal Stair Ascent and Descent. J. Biomech..

[6] Wang L., Ma W., Zhu W., Zhai L., Sun Y. (2025). Effects of Experimentally Induced Lower Limb Muscle Fatigue on Healthy Adults’ Gait: A Systematic Review. Bioengineering.

[7] Vøllestad N.K. (1997). Measurement of Human Muscle Fatigue. J. Neurosci. Methods.

[8] Swaen G.M.H., Van Amelsvoort L.G.P.M., Bültmann U., Kant I.J. (2003). Fatigue as a Risk Factor for Being Injured in an Occupational Accident: Results from the Maastricht Cohort Study. Occup. Environ. Med..

[9] Wang L., Ma W., Zhu W., Xie Q., Sun Y. (2025). Impact of Lower-Limb Muscle Fatigue on Dynamic Postural Control During Stair Descent: A Study Using Stair-Embedded Force Plates. Sensors.

[10] Yu C., Zhan J., Xu L., Zhou J., Fu W. (2025). Motor Control Performance-Related Modulation of Beta-Band EEG–sEMG Coherence Differs between General and Local Muscular Exercise-Induced Fatigue. Eur. J. Appl. Physiol..

[11] Hsiao H., Simeonov P. (2001). Preventing Falls from Roofs: A Critical Review. Ergonomics.

[12] Parijat P., Lockhart T.E. (2008). Effects of Lower Extremity Muscle Fatigue on the Outcomes of Slip-Induced Falls. Ergonomics.

[13] Granacher U., Gruber M., Förderer D., Strass D., Gollhofer A. (2010). Effects of Ankle Fatigue on Functional Reflex Activity during Gait Perturbations in Young and Elderly Men. Gait Posture.

[14] Hamacher D., Törpel A., Hamacher D., Schega L. (2016). The Effect of Physical Exhaustion on Gait Stability in Young and Older Individuals. Gait Posture.

[15] Lipscomb H.J., Glazner J.E., Bondy J., Guarini K., Lezotte D. (2006). Injuries from Slips and Trips in Construction. Appl. Ergon..

[16] Parijat P., Lockhart T.E. (2008). Effects of Quadriceps Fatigue on the Biomechanics of Gait and Slip Propensity. Gait Posture.

[17] Hunt M.A., Hatfield G.L. (2017). Ankle and Knee Biomechanics during Normal Walking Following Ankle Plantarflexor Fatigue. J. Electromyogr. Kinesiol..

[18] Qu X. (2015). Effects of Lower-Limb Muscular Fatigue on Stair Gait. J. Biomech..

[19] Kang H. (2021). Sample Size Determination and Power Analysis Using the G*Power Software. J. Educ. Eval. Health Prof..

[20] van Melick N., Meddeler B.M., Hoogeboom T.J., der Sanden M.W.G.N., van Cingel R.E.H. (2017). How to Determine Leg Dominance: The Agreement between Self-Reported and Observed Performance in Healthy Adults. PLoS ONE.

[21] Lu Z., Mao C., Tan Y., Liu T., Li X., Li Z., Zhu W., Sun Y. (2024). The Impact of Backpack Load on Adolescent’s Stair Descent Gait. J. Biomech..

[22] Hinman R.S., Bennell K.L., Metcalf B.R., Crossley K.M. (2002). Delayed Onset of Quadriceps Activity and Altered Knee Joint Kinematics during Stair Stepping in Individuals with Knee Osteoarthritis. Arch. Phys. Med. Rehabil..

[23] Pincivero D.M., Gandhi V., Timmons M.K., Coelho A.J. (2006). Quadriceps Femoris Electromyogram during Concentric, Isometric and Eccentric Phases of Fatiguing Dynamic Knee Extensions. J. Biomech..

[24] Yaggie J.A., McGregor S.J. (2002). Effects of Isokinetic Ankle Fatigue on the Maintenance of Balance and Postural Limits. Arch. Phys. Med. Rehabil..

[25] Cheng A.J., Rice C.L. (2005). Fatigue and Recovery of Power and Isometric Torque Following Isotonic Knee Extensions. J. Appl. Physiol..

[26] Murdock G.H., Hubley-Kozey C.L. (2012). Effect of a High Intensity Quadriceps Fatigue Protocol on Knee Joint Mechanics and Muscle Activation during Gait in Young Adults. Eur. J. Appl. Physiol..

[27] Sun Y., Wei S., Zhong Y., Fu W., Li L., Liu Y. (2015). How Joint Torques Affect Hamstring Injury Risk in Sprinting Swing-Stance Transition. Med. Sci. Sports Exerc..

[28] Yang C., Bouffard J., Srinivasan D., Ghayourmanesh S., Cantú H., Begon M., Côté J.N. (2018). Changes in Movement Variability and Task Performance during a Fatiguing Repetitive Pointing Task. J. Biomech..

[29] Jensen J.S., Nielsen J.L., Sorensen A.S., Aagaard P., Holsgaard-Larsen A., Bojsen-Moller J. (2025). Kinematic and Kinetic Analysis of Sit-to-Stand and Stair-Walking with Dynamic Robot-Assisted Body Weight Unloading. J. Biomech..

[30] Pataky T.C. (2012). One-Dimensional Statistical Parametric Mapping in Python. Comput. Methods Biomech. Biomed. Engin..

[31] Prilutsky B.I., Herzog W., Leonard T.R., Allinger T.L. (1996). Role of the Muscle Belly and Tendon of Soleus, Gastrocnemius, and Plantaris in Mechanical Energy Absorption and Generation during Cat Locomotion. J. Biomech..

[32] Vila-Chã C., Riis S., Lund D., Møller A., Farina D., Falla D. (2011). Effect of Unaccustomed Eccentric Exercise on Proprioception of the Knee in Weight and Non-Weight Bearing Tasks. J. Electromyogr. Kinesiol. Off. J. Int. Soc. Electrophysiol. Kinesiol..

[33] Ronai P., Gallo P.M. (2020). The Stair Climb Power Test. Acsm’s Health Fit. J..

[34] Neptune R.R., Zajac F.E., Kautz S.A. (2004). Muscle Mechanical Work Requirements during Normal Walking: The Energetic Cost of Raising the Body’s Center-of-Mass Is Significant. J. Biomech..

[35] Lin Y.-C., Fok L.A., Schache A.G., Pandy M.G. (2015). Muscle Coordination of Support, Progression and Balance during Stair Ambulation. J. Biomech..

[36] Moniz-Pereira V., Kepple T.M., Cabral S., João F., Veloso A.P. (2018). Joint Moments’ Contributions to Vertically Accelerate the Center of Mass during Stair Ambulation in the Elderly: An Induced Acceleration Approach. J. Biomech..

[37] Kellis E., Kouveliod V. (2009). Agonist versus Antagonist Muscle Fatigue Effects on Thigh Muscle Activity and Vertical Ground Reaction during Drop Landing. J. Electromyogr. Kinesiol..

[38] Padua D.A., Arnold B.L., Perrin D.H., Gansneder B.M., Carcia C.R., Granata K.P. (2006). Fatigue, Vertical Leg Stiffness, and Stiffness Control Strategies in Males and Females. J. Athl. Train..

[39] Farris D.J., Sawicki G.S. (2011). The Mechanics and Energetics of Human Walking and Running: A Joint Level Perspective. J. R. Soc. Interface.

[40] Barbieri F.A., Gobbi L.T.B., Lee Y.J., Pijnappels M., van Dieen J.H. (2014). Effect of Triceps Surae and Quadriceps Muscle Fatigue on the Mechanics of Landing in Stepping down in Ongoing Gait. Ergonomics.

[41] Lu Y., Wu Y., Liu Z., Ren S. (2024). Research Status of Gait Biomechanical Characteristics after Anterior Cruciate Ligament Rupture and Reconstruction. Acta Sci. Nat. Univ. Pekin..

[42] Kallini J.R., Fowler E.G., Pietruszewski L., Vuong A., Greenberg M.B., Jackson N.J., Thompson R., Bernthal N., Stearns-Reider K. (2025). Gait and Health-Related Quality of Life Outcomes Following Proximal Femoral Tumor Resection and Reconstruction with Tensioning of the Abductor Musculotendinous Unit. Clin. Biomech..

